# Mössbauer and X-ray Studies of Radiation-Induced Processes in Nb–Zr Alloys Implanted with ^57^Fe Ions

**DOI:** 10.3390/ma16103813

**Published:** 2023-05-18

**Authors:** Mikhail Vereshchak, Irina Manakova, Adilkhan Shokanov

**Affiliations:** 1 Institute of Nuclear Physics, Ibragimov St. 1, Almaty 050032, Kazakhstan; 2 Institute of Mathematics, Physics and Informatics, Abai Kazakh National Pedagogical University, Dostyk Av. 13, Almaty 050010, Kazakhstan

**Keywords:** Nb–Zr alloy, implantation, Mössbauer spectroscopy, radiation damage

## Abstract

The effect of implanting ^57^Fe ions on the crystal structure of Nb–Zr alloys has been studied using Mössbauer spectroscopy on ^57^Fe nuclei and X-ray diffraction. As a result of implantation, a metastable structure was formed in the Nb–Zr alloy. The XRD data indicated a decrease in the crystal lattice parameter of niobium; that is, there was a compression of the niobium planes when implanted with iron ions. Mössbauer spectroscopy revealed three states of iron. The singlet indicated a supersaturated Nb(Fe) solid solution; the doublets characterized the diffusion migration of atomic planes and crystallization of voids. It was shown that the values of the isomer shifts in all three states did not depend on the implantation energy, which indicates the invariance of the electron density on the ^57^Fe nuclei in the studied samples. The resonance lines of the Mössbauer spectra were significantly broadened, which is typical for materials with low crystallinity and a metastable structure that is stable at room temperature. The paper discusses the mechanism of radiation-induced and thermal transformations in the Nb–Zr alloy, which leads to the formation of a stable well-crystallized structure. A Fe_2_Nb intermetallic compound and the Nb(Fe) solid solution formed in its near-surface layer, while Nb(Zr) remained in the bulk.

## 1. Introduction

Developing structural materials for operating and prospective nuclear plants is a very complex scientific and technical task [1,2,3].

Niobium alloys started to be developed in the early 1950s when it became necessary to create high-temperature materials for new technologies (rocket science, aviation and space technology, nuclear energy, etc.). Niobium alloys are characterized by good technological properties, a relatively low density, low thermal neutron capture cross-section and good resistance in contact with liquid metal coolants. Almost all niobium alloys have high hardness and electrical conductivity. These alloys are widely used as high-temperature fuel cells. They have good stability against mechanical and radiation damage. Zr additives are introduced into Nb-based alloys to increase strength and improve oxidation resistance and compatibility with liquid metals [4,5]. Only 1% of Zr in the alloy composition is sufficient to provide flexibility and toughness, making it suitable for the production of the thinnest wires and foils.

Nb–Zr alloys, used in modern nuclear reactors, contain a niobium concentration from 2% to 10% by weight. The composition of these alloys is carefully controlled to ensure optimum properties for certain types of nuclear reactor applications, including good mechanical strength, corrosion resistance and low neutron absorption. Zr–2.5Nb alloys are used in pressurized water reactors (PWRs) and boiling water reactors (BWRs); Zr–2Nb alloys are utilized in CANDU reactors; and Zr–3Nb alloys are used in research reactors. Zr–Nb alloys are also used as cladding materials for light water reactors (LWRs) [6]. Nb–Zr alloys with a Nb content of up to 10 wt% have demonstrated excellent properties, mainly resistance to corrosion and creeping under irradiation [7,8]. Investigations of Nb–Zr composites containing up to 20 wt% Nb were completed in [9,10,11].

Medium-entropy NbZrTi alloys and high-entropy refractory NbZrTi-based alloys have shown great mechanical performances and great opportunities for applications as high-temperature structural materials [12,13,14].

Nb alloys with a low Zr content are also required at nuclear facilities; in particular, Nb–1Zr niobium pipe is used in pipeline construction and pumping equipment. In addition, such alloys were considered for the design of compact high-temperature reactors (HTRs) [4,5,15], which operate under the conditions of aggressive media, high temperatures, irradiation and possible exposure to the atmosphere at high temperatures.

The paper [16] provides a discussion of possible applications of Nb and Nb–1Zr alloys in structural element production for generation IV fission reactors.

One of the requirements for structural materials is radiation resistance, which is closely related to their mechanical properties. It was shown in [6,17,18,19,20,21] that ion implantation of a wide range of atomic ions in charged particle accelerators is a convenient method to investigate the radiation resistance of materials using neutron irradiation simulations. It is known that ion implantation can produce compounds that are not present in equilibrium state diagrams. The final structural-phase state of materials subjected to ion implantation depends on many factors: elemental composition of the initial material, implantation temperature, fluence, implant energy and current density, which are very important for selecting the optimal, economically justified implantation conditions.

Mössbauer spectroscopy, X-ray diffraction (XRD), scanning electron microscopy (SEM), electron microanalysis [22] and the pseudo-potential density functional theory (PP-DFT) method [23] were used to study ternary Zr–Nb–Fe alloys corresponding in composition to the middle part of the equilibrium state diagram of the system. According to the results, it was concluded that Fe in the two Laves phases, present in the Zr–Nb–Fe system, forms two types of bcc of β-phases (Zr-enriched and Nb-rich). Binary Nb–Fe systems were studied in [24,25,26]. In addition to two solid solutions at the edges of the Nb–Fe binary diagram, the hyperfine interactions of intermetallic phases Fe_2_Nb (ε-phase) and Fe_7_Nb_6_ (μ-phase) parameters were determined. The non-magnetic components of the spectra [25] indicated the presence of several crystalline phases Fe_2_Nb, FeNb, a solid solution of Fe in Nb, and amorphous phases of Fe_x_Nb_100-x_ in the concentration range of 20 < x < 80. It should be noted that nuclear gamma-resonance spectroscopy is not always a widely used method in similar works; however, several papers [27,28,29,30,31] showed that this method provides abundant information that is not available using other research methods.

The physical processes that occur during the implantation of ^57^Fe ions, which are typical for all materials subjected to irradiation, include sputtering of atoms of the surface layer, formation and dissolution of phases, and ion mixing [28,32,33]. The formation of strongly heated regions is also possible, with their subsequent hardening leading the irradiated materials to amorphization. The diffusion processes in solids between substances A and B are described by different partial diffusion coefficients of the components D_A_ and D_B_ (D_A_ > D_B_) and result in two phenomena. These phenomena are called the Kirkendall and Frenkel effects. The motion of the interface between substances A and B shifts towards substance A—the Kirkendall effect. The absorption of excess vacancies by introduced inhomogeneities leads to the formation of macroscopic pores in substance A—the Frenkel effect.

When materials are irradiated, the predominant mechanism for the formation of structural-phase damage is through cascades of atom–atom collisions. The cascades cover significant areas of the crystal and generate a large number of substitutions and large vacancy clusters along the periphery of which interstitial atoms are located [34]. Further, during this time, a ~10^−13^ s evolution follows involving the annealing of defects, i.e., the process of healing the depleted zone. In this case, the segregation of atoms occurs in displacement cascades, resulting in the formation of regions enriched in one of the alloy components. Thus, in [34], during the study of a layered Be–Fe–Be system, it was established that irradiation with He ions resulted in the transition of some Fe atoms from a magnetically ordered to a paramagnetic state (the effect of radiation-induced segregation in the zone of primary knocked-out atoms). Such processes were also observed in [35], and similar results were obtained in [28].

The purpose of this research is to study the radiation-induced processes in 2.1Zr alloys implanted with ions of the ^57^Fe Mössbauer atom and to obtain data on the effect of the zone of influence of these ions on the structural-phase state of the implanted samples. The elemental composition of the alloy chosen for this research belongs to the insufficiently studied niobium angle of the state diagram of the Nb–Zr binary system.

## 2. Materials and Methods

The samples for our study were prepared from Nb and Nb–Zr alloy plates rolled to an effective thickness (20 μm). The resulting foils were subjected to homogenizing annealing at a temperature of 800 °C in a vacuum pressure of 5 × 10^−7^ mm Hg for 2 h.

The implantation of high-energy singly charged ^57^Fe ions with an energy of 1 MeV into Nb (sample A0) and ^57^Fe ions with energies of 1, 0.35 and 0.25 MeV into the Nb–Zr alloy (samples A1, A2, A3, respectively) with a fluence of 5 × 10^16^ ion/cm^2^ and an ion beam current of ~100 nA was performed using the heavy ion accelerator UKP-2-1 at the Institute of Nuclear Physics (Almaty, Kazakhstan). The target chamber was additionally equipped with a magnetic discharge pump, which made it possible to obtain a vacuum pressure of ~1.5 × 10^−7^ mm Hg and to minimize the rate of carbon film burning on the target during implantation. The target temperature under the selected irradiation modes did not exceed 60 °C.

The elemental composition of the studied samples was determined using a scanning electron microscope JEOLJSM-06610 (Tokyo, Japan) equipped with an IncaX-act energy dispersive analyzer with a resolution of 3 nm at an accelerating voltage of 0.3–30 kV and accumulation time of 10 min. Before implantation, the Nb–Zr alloy contained 97.9% Nb and 2.1% Zr.

The changes in the crystal structure of the studied materials before and after implantation were recorded using a diffractometer Bruker D8 ADVANCE (Karlsruhe, Germany) with a Cu-K_α_ emitter in the Bragg-Brentano geometry. Crystalline phases were identified using the file cabinet ASTM and TCPDS.

**Figure 1:**
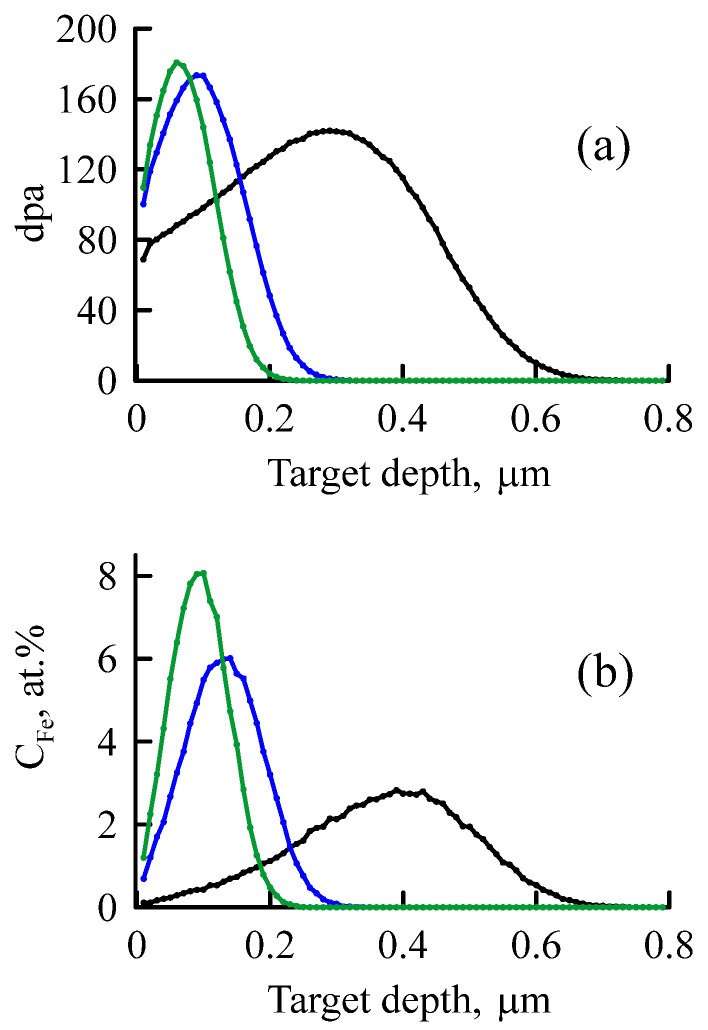
Damage profiles (**a**) and ^57^Fe ion concentration profiles (**b**) during implantation of the Nb–Zr alloy with ions energies of 1 MeV (black), 0.35 MeV (blue), 0.25 MeV (green).

The SRIM-2008 program [36,37] in Quick Calculation mode was used to assess the degree of ion beam impact on the crystal lattice of the implanted materials. The average projective range of ions and the number of displacements per atom (dpa) were determined. The concentration of implanted ^57^Fe ions in the target and the concentration of vacancies and knocked-out atoms in the matrix were calculated; the number of backscattered ^57^Fe ions was estimated. With a decrease in the implantation energy, the number of displacements per atom and ^57^Fe concentration increased (Figure 1).

The electronic environment of ^57^Fe atoms after completion of the implantation process was determined using Mössbauer spectroscopy in transmission geometry (MS) mode and in the electron channel backscattering mode (CEMS). Mössbauer studies were performed using a MS-1104Em spectrometer at room temperature. ^57^Co in a chromium matrix served as the source of γ-quanta; gamma radiation was recorded using a resonant scintillation detector. Conversion electrons were recorded using a gas-proportional counter with a He + 8% CH_4_ mixture. The Mössbauer spectra were processed through model interpretation using the SpectrRelax program [38].

## 3. Results

**Figure 2:**
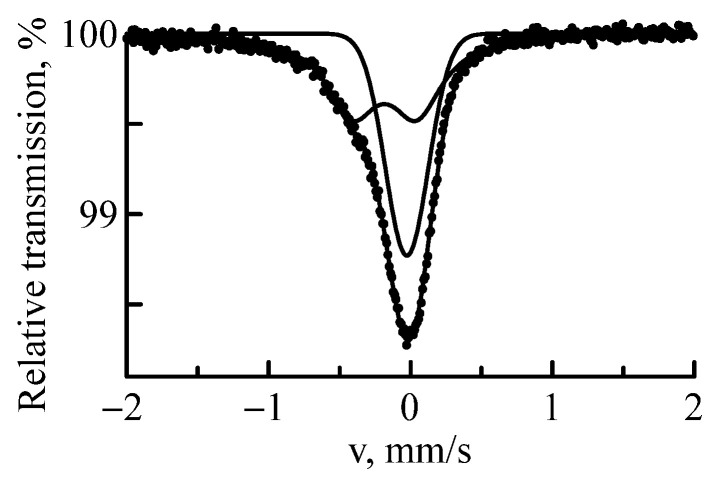
MS spectrum of ^57^Fe nuclei in the Nb sample subjected to implantation of ^57^Fe ions with 1 MeV energy.

Figure 2 shows the MS spectrum of Nb implanted with ^57^Fe ions with a fluence of 5 × 10^16^ ion/cm^2^ and 1 MeV energy. The sample spectrum consisted of a singlet and a doublet. Similar results were obtained earlier when ^57^Fe ions were implanted into one-component materials (Ta, Mo, Al, Zr) [20,32,39,40]. Isomer shifts and quadrupole splittings are typical and specific for these metals with ^57^Fe probe atoms. Thus, it can be stated with a high probability that in the spectrum of ^57^Fe implanted in Nb, the singlet with an isomer shift of δ = −0.026 mm/s is inherent in the implants localized at the sites of the cubic crystal lattice of the target [30,32] in a volume limited by the projective range of the ions. It is known that elements of the 3d group have a low solubility when alloyed with Nb (at 600 °C, the solubility of Fe in Nb is only 0.1 at.%). It should be assumed that the observed singlet can be interpreted as a solid solution of Fe substitution in Nb–Nb(Fe). Under conditions of intensive implantation, the limiting solubility can increase significantly. Indeed, in the implanted Nb layer (3.591 × 10^−5^ cm), 5 × 10^16 57^Fe ions were localized, 46% of which were in a solid solution Nb(Fe) (see Table 1). In addition to the singlet, the spectrum contained a doublet with an isomer shift of δ = −0.18 mm/s and a quadrupole splitting of Δ = 0.45 mm/s. The singlet and the doublet were defined by ^57^Fe in the paramagnetic state. This indicates that the implant was atomically scattered over the target volume and did not have Mössbauer atoms in the nearest environment. The ions of ^57^Fe in the singlet and doublet had a negative isomer shift and, as a consequence, a higher electron density relative to metallic iron.

**Figure 3:**
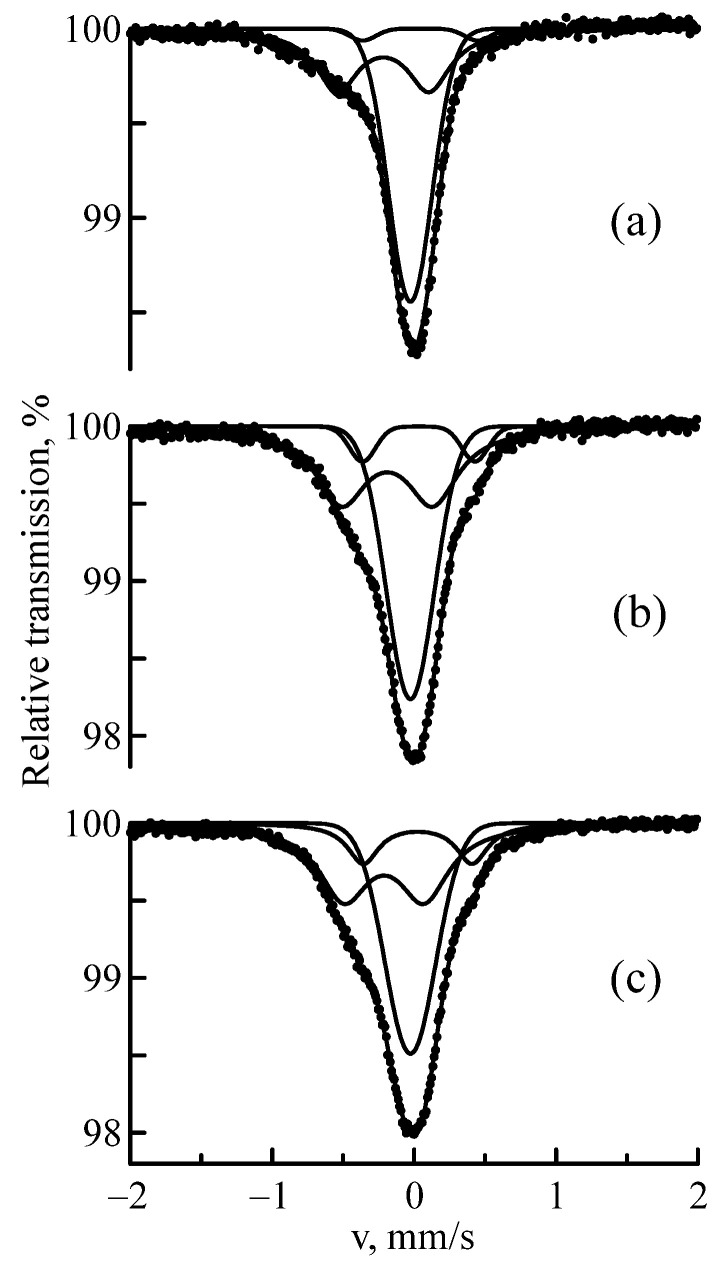
MS spectra of ^57^Fe nuclei in the Nb–Zr alloy samples subjected to implantation of ^57^Fe ions with 1 (**a**), 0.35 (**b**), and 0.25 (**c**) MeV energies.

Figure 3 shows the MS spectra of the Nb–Zr alloy subjected to implantation of ^57^Fe ions with a fluence of 5 × 10^16^ ion/cm^2^ and energies of 1, 0.35 and 0.25 MeV. The MS spectra were processed with sufficient quality using two doublets and a singlet; the Mössbauer parameters are provided in Table 1.

The distribution of ^57^Fe ions (N_i_) over the individual phases of the samples (Table 2) was calculated according to the MS results. The following formula was used for the calculation:

N_i_ = D·S (1 − Y) I_i_/100, (1)

where D is the fluence;

S is the area of the target under the beam;

Y is the backscattering coefficient;

I_i_ is the relative spectral area of the i phase.

It should be noted that at the chosen energies, the backscattering coefficient Y obtained from the SRIM program is very small, so this parameter was not taken into account in further discussions. The number of backscattered ^57^Fe ions increased with decreasing implant energy from 2.05 × 10^14^ at 1.0 MeV energy (Y = 0.004) to 7.45 × 10^14^ at 0.25 MeV energy (Y = 0.015).

**Table 2:** Distribution of ^57^Fe ions over phases of the Nb–Zr alloy samples subjected to implantation of ^57^Fe ions with 1, 0.35 and 0.25 MeV energies.

Sample	Singlet, Ions	Doublet 1, Ions	Doublet 2, Ions
A0	2.30 × 10^16^	2.70 × 10^16^	
A1	2.80 × 10^16^	2.05 × 10^16^	0.15 × 10^16^
A2	2.65 × 10^16^	1.95 × 10^16^	0.40 × 10^16^
A3	2.50 × 10^16^	1.80 × 10^16^	0.70 × 10^16^

Analysis of the Mössbauer spectra suggests that the ^57^Fe ions in the samples (Singlet and Doublet 1) had a charge density on the nucleus of +4 with an electronic configuration of the outer shells of 3s^2^3p^6^3d^4^4s^0^. The isomer shift of the Singlet was less than that of α-Fe. Consequently, the electron density on the ^57^Fe nucleus, a substitutional impurity in the Nb crystal lattice, was greater. The isomer shift of Doublet 1 was also smaller than that of the α-Fe and Singlet, which is associated with a strong disordering of the crystal lattice, disorder, partial amorphization and the growth of the electron density on the ^57^Fe nucleus. It is known that the isomer shift is determined by the electron density on the nucleus and is directly related to the nature of interaction in the crystal. The change in compounds allows us to obtain information about the redistribution of electrons between the atoms of the components and the change in the strength of interatomic bonds in the crystal lattice. The charge density on the nucleus is only created by s-electrons. Even though d-electrons have zero density in the nucleus, their shielding effect on s-electrons contributes to the total electron density. The growth of d-electrons density in an atom reduces the charge density on the nucleus and leads to a positive isomer shift relative to α-Fe. The isomer shift of Doublet 2 was greater than that of α-Fe. The value of the isomer shift and quadrupole splitting of this phase indicates that Fe had a charge density of +3, and the electron configuration of the outer shells was 3s^2^3p^6^3d^5^4s^0^. The implant was localized in disordered amorphous areas of the target and had a reduced charge electron density. This is possible due to the shielding of the outer 3s-electrons by additional electrons of the d-shell. The relative area of Doublet 2 was small at high implantation energies and increased with decreasing implantation energy.

Since the diffusion coefficients of the alloy components are different, the corresponding atomic fluxes are also different. Of the two counter streams, the one that is directed from the crystal with a lower melting point is larger. Thus, in a region with high flux, vacancies are formed at the lattice sites. It is worth mentioning that in the process of mutual diffusion, vacancies can appear and disappear without a trace on the sinks of dislocations, cracks, pores, etc. Similar phenomena cannot occur with atoms, as their numbers are constant. Consequently, as a result of diffusion processes, not only does relocation of plane atoms occur, but also the growth of voids. Two types of sinks are formed: formation of the vacancies (voids) and relocation of the atomic planes [41].

**Figure 4:**
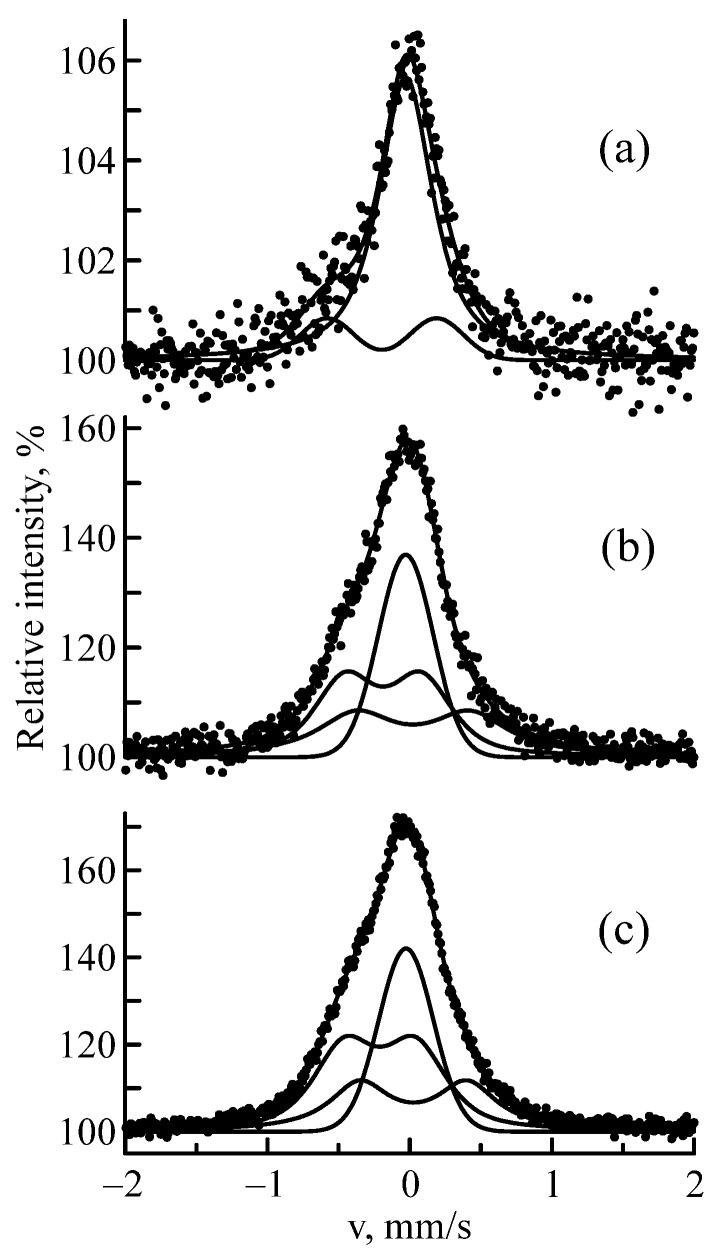
CEMS spectra of ^57^Fe nuclei in the Nb–Zr alloy samples subjected to implantation of ^57^Fe ions with 1 (**a**), 0.35 (**b**), and 0.25 (**c**) MeV energies.

The amorphous structure was more evident in the CEMS spectra of the samples implanted with ^57^Fe ions with lower energies. The highest implant concentration was observed at these energies (see Figure 1), which causes a decrease in crystallinity and an increase in amorphism of the Nb–Zr alloy. Figure 4 shows the CEMS spectra of the Nb–Zr alloy samples subjected to implantation of ^57^Fe ions with 0.25, 0.35 and 1 MeV energies; the Mössbauer parameters are indicated in Table 3. An increase in the resonance lines widths indicates a highly amorphous structure.

The elemental composition of the samples after implantation of ^57^Fe ions was determined using SEM. The analysis was performed on 10 points and was calculated as an average value. Sample A2 contained 96.89% Nb, 1.58% Zr and 1.53% Fe; sample A3 contained 96.65% Nb, 1.61% Zr and 1.74% Fe.

The changes in the crystal structure of the studied materials before and after the implantation of ^57^Fe ions were identified using XRD. X-ray diffraction patterns of Nb (JCPDS card No. 35-0789) and Nb–Zr alloy samples before irradiation were represented by three reflections. However, the reflections in the Nb–Zr alloy were shifted towards smaller angles. This means that Zr as an impurity was localized at the sites of the Nb crystal lattice. The atomic radius of this impurity (1.60) was larger than that of Nb (1.43). This led to an increase in the lattice parameter of the Nb–Zr alloy (a = 3.315 Å) in comparison with the lattice parameter of Nb (a = 3.301 Å).

**Figure 5:**
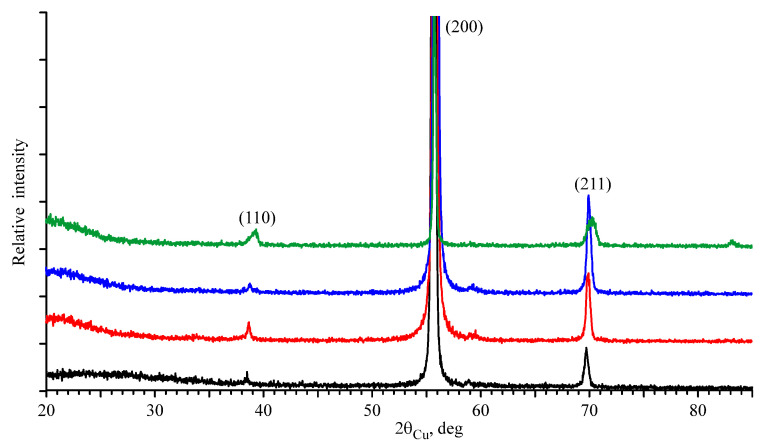
X-ray diffraction patterns of the Nb–Zr alloy samples before implantation (black) and after implantation with ^57^Fe ions with 1 MeV (red), 0.35 MeV (blue), 0.25 MeV (green) energies.

The XRD pattern of the Nb–Zr alloy before and after implantation of ^57^Fe ions with energies of 1, 0.35 and 0.25 MeV are shown in Figure 5. After irradiation, the reflections in the Nb–Zr alloy were shifted towards larger angles, indicating an out-of-plane compressive strain [18]. It can be concluded that after irradiation, the system tends to relax, i.e., the atomic planes tend to move to be similar to Nb; a similar behavior was observed after Cu irradiation of Zr/Nb [6,17]. In the zone of primary knocked-out atoms, there was a depletion of zirconium and niobium atoms and an enrichment of iron ions, i.e., radiation-induced segregation occurs in the region of the solid solution Fe in the Nb–Zr alloy. This led to a decrease in the crystal lattice parameter (see Table 4). ^57^Fe localized in the cubic Nb crystal lattice corresponded to a singlet in the MS spectrum. Broadened resonance lines of the doublets, detected by nuclear gamma-resonance spectroscopy, are typical for materials with low crystallinity and high amorphism. Such phases are not amenable to XRD, but their presence is noticeable on the background track of the X-ray diffraction patterns. The broadening and asymmetry of the diffraction peaks of the A3 sample can be explained by the higher amorphism of the irradiated sample. This is associated with a lower projective range of ^57^Fe ions, which leads to an increase in the concentration of the implant in the surface layer.

In this paper, the interpretation of the obtained experimental results was based on the mechanisms of the Kirkendall and Frenkel effects. Diffusion migration of atomic planes from crystal to crystal is reflected in Doublet 1; crystallization of the void, along the periphery of which the knocked-out atoms are located, is reflected in Doublet 2. These mechanisms were previously used to explain the segregation of atoms in displacement cascades in the zone of primary knocked-out atoms [28,34,35]. In the zone of primary knocked-out atoms, strongly heated regions are formed (thermal peaks), which can be compared with atomic plasma. When this plasma cools at a rapid rate (10^10^–10^15^ K/s), it hardens with a transition of the irradiated medium into a metastable amorphous state.

**Figure 6:**
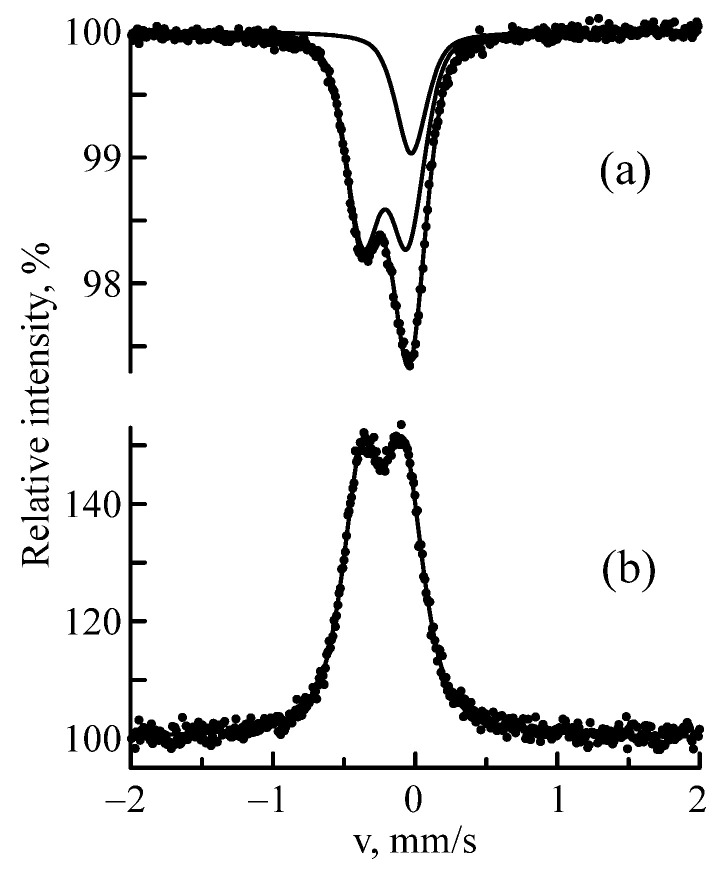
MS (**a**) and CEMS spectra (**b**) of ^57^Fe nuclei in the Nb–Zr alloy sample subjected to implantation of ^57^Fe ions with 0.25 MeV energy after annealing at 1000 °C.

As noted above, the most sensitive effect of irradiation was displayed in the Mössbauer spectra of the Nb–Zr alloy sample after implantation with ^57^Fe ions with 0.25 MeV energy. Therefore, the crystallization kinetics of this sample was studied. After irradiation, Sample A3 was subjected to successive four-hour isochronous annealings in a vacuum pressure of 5 × 10^−7^ mm Hg at temperatures up to 1000 °C with a step of 200 °C. The most typical MS and CEM spectra of this sample are shown in Figure 6; the parameters of hyperfine interactions are indicated in Table 5. It can be seen that the spectra of the annealed sample sharply changed in structure. Doublet 2 had completely disappeared. An explanation for this can be found using the inverse Kirkendall effect.

The vacancy flow causes a depletion of the sink region in components with a high diffusion rate (Fe) and enrichment in components with a lower diffusion coefficient (Zr). The intensity of the singlet in the MS spectrum of sample A3 before annealing was 45%, and in the CEMS spectrum, it was 32%. Sample annealing at 1000 °C led to a decrease in intensity (up to 23%) in the MS spectrum and to the complete disappearance of this component in the CEMS spectrum. The explanation for this fact lies in the results of the recrystallization annealing of Nb(Fe). There are two mechanisms involved in the implantation process. As a result of radiation-induced segregation, a new phase is released on the surface and on the defects in the crystal lattice (dislocations, pores, grain boundaries, etc.) of the irradiated material. In addition, during radiation-induced separation, a similar phase is also nucleated in the region of the crystal that does not contain extended defects [41]. This is associated with the fact that the regions remaining at the site of the atom–atom collision cascade passage has a structure typical of the point defect distribution—a zone depleted in atoms in the center with interstitial atoms located along the periphery. Moreover, a situation is possible when the depleted zone will be replenished with atoms predominantly of the same type. Fe combines with the matrix element (Nb), forming the Fe_2_Nb intermetallic compound, the Mössbauer parameters of which are similar to those of Doublet 1 and the intermetallic compound obtained by mechanical alloying [42,43]. The zirconium atoms return to their primary state, forming the Nb(Zr) solid solution. This was confirmed by the increase in the crystal lattice parameter of the annealed Nb–Zr alloy sample (a = 3.317 Å).

A layered system was produced as a result of the studies of the alloy subjected to ion implantation and subsequent thermal annealing. According to the Mössbauer spectroscopy results, it was established that the layer to a depth of 1 × 10^−5^ cm was represented by Fe_2_Nb intermetallic compound. Further, down to the depth of the possible projective range of ^57^Fe ions, the layer was characterized by a binary structure consisting of the Fe_2_Nb intermetallic compound and the Nb(Fe) solid solution with a limiting solubility of 0.1 at.% Fe. According to the XRD data, the basis of the sample was the Nb(Zr) solid solution.

## 4. Conclusions

The Nb–Zr alloy was implanted with ^57^Fe ions with 1, 0.35 and 0.25 MeV energies and a fluence of 5 × 10^16^ ion/cm^2^. The electronic state of ^57^Fe and the crystal structure of the implanted samples were determined using Mössbauer spectroscopy on ^57^Fe nuclei and X-ray diffraction. As a result of implantation, a metastable structure was formed in the Nb–Zr alloy. The XRD data indicated a decrease in the crystal lattice parameter of niobium; that is, there is a compression of the niobium planes implanted with iron ions. MS and CEMS spectroscopy revealed three states of iron. The singlet indicated a supersaturated Nb(Fe) solid solution; Doublet 1 characterized the diffusion migration of atomic planes; Doublet 2 indicated the crystallization of voids. It was shown that the values of the isomer shifts in all three states did not depend on the implantation energy, which indicates the invariance of the electron density on the ^57^Fe nuclei in the studied samples. The resonance lines of the Mössbauer spectra were significantly broadened, which is typical for materials with a low crystallinity and metastable structure that is stable at room temperature. The amorphous structure was more evident in the CEMS spectra of the samples implanted with low-energy ^57^Fe ions. A stable structure was obtained by annealing the implanted alloy at 1000 °C. It consisted of a highly crystallized Nb(Fe) solid solution with a limiting solubility of 0.1 at.% Fe and a well-crystallized Fe_2_Nb intermetallic compound.

## Figures and Tables

**Table 1:** Hyperfine parameters of the MS spectra of the Nb and Nb–Zr alloy samples.

Sample	Singlet			Doublet 1				Doublet 2			
	δ, mm/s	W, mm/s	I, %	δ, mm/s	Δ, mm/s	W, mm/s	I, %	δ, mm/s	Δ, mm/s	W, mm/s	I, %
A0	−0.026	0.36 ± 0.01	46 ± 2	−0.18 ± 0.01	0.45 ± 0.01	0.44 ± 0.01	54 ± 2				
A1	−0.026	0.36 ± 0.01	59 ± 2	−0.22 ± 0.01	0.65 ± 0.01	0.37 ± 0.01	38 ± 2	0.04 ± 0.01	0.81 ± 0.01	0.22 ± 0.01	3 ± 2
A2	−0.026	0.40 ± 0.01	50 ± 2	−0.19 ± 0.01	0.64 ± 0.01	0.44 ± 0.02	43 ± 2	0.03 ± 0.01	0.79 ± 0.01	0.22 ± 0.01	7 ± 2
A3	−0.026	0.42 ± 0.02	45 ± 2	−0.21 ± 0.01	0.56 ± 0.01	0.43 ± 0.01	41 ± 1	0.03 ± 0.01	0.77 ± 0.01	0.27 ± 0.01	14 ± 2

δ: isomer shift relative to metallic iron at 300 K; Δ: quadrupole splitting; W: line width; I: relative spectral area.

**Table 3:** Hyperfine parameters of the CEMS spectra of the Nb and Nb–Zr alloy samples.

Sample	Singlet			Doublet 1				Doublet 2			
	δ, mm/s	W, mm/s	I, %	δ, mm/s	Δ, mm/s	W, mm/s	I, %	δ, mm/s	Δ, mm/s	W, mm/s	I, %
A0	−0.026	0.60 ± 0.04	100								
A1	−0.026	0.53 ± 0.02	94 ± 3	−0.20 ± 0.02	0.50 ± 0.01	0.27 ± 0.05	6 ± 3				
A2	−0.026	0.46 ± 0.02	34 ± 2	−0.19 ± 0.02	0.54 ± 0.01	0.50 ± 0.03	36 ± 3	0.03 ± 0.01	0.80 ± 0.02	0.65 ± 0.02	30 ± 4
A3	−0.026	0.46 ± 0.03	32 ± 2	−0.21 ± 0.01	0.50 ± 0.01	0.51 ± 0.02	41 ± 1	0.03 ± 0.01	0.75 ± 0.02	0.51 ± 0.02	27 ± 3

**Table 4:** Crystal lattice parameter ***a*** of the Nb–Zr alloy samples after implantation of ^57^Fe ions with 1, 0.35 and 0.25 MeV energies.

Sample	*a*, (Å)
A1	3.303
A2	3.298
A3	3.280

**Table 5:** Hyperfine parameters of the MS and CEMS spectra of the Nb–Zr alloy sample subjected to implantation of ^57^Fe ions with 0.25 MeV energy after annealing at 1000 °C.

Sample	Singlet			Doublet			
	δ, mm/s	W, mm/s	I, %	δ, mm/s	Δ, mm/s	W, mm/s	I, %
MS	−0.026	0.26 ± 0.01	23 ± 1	−0.21 ± 0.01	0.31 ± 0.01	0.29 ± 0.01	77 ± 1
CEMS				−0.23 ± 0.01	0.30 ± 0.01	0.32 ± 0.01	100

## Data Availability

The data presented in this study are available on request from the corresponding author.
